# Collaborative Activities of Noradrenaline and Natriuretic Peptide for Glucose Utilization in Patients with Acute Coronary Syndrome

**DOI:** 10.1038/s41598-019-44216-0

**Published:** 2019-05-24

**Authors:** Goki Uno, Tomohisa Nagoshi, Akira Yoshii, Yasunori Inoue, Yoshiro Tanaka, Haruka Kimura, Satoshi Ito, Kazuo Ogawa, Toshikazu D. Tanaka, Kosuke Minai, Takayuki Ogawa, Makoto Kawai, Michihiro Yoshimura

**Affiliations:** Division of Cardiology, Department of Internal Medicine, The Jikei University School of Medicine, Tokyo, Japan

**Keywords:** Unstable angina, Myocardial infarction

## Abstract

Glucose is an important preferential substrate for energy metabolism during acute coronary syndrome (ACS) attack, although insulin resistance (IR) increases during ACS. Increasing evidence indicates that natriuretic peptides (NP) regulate glucose homeostasis. We investigated possible compensatory actions of NP in collaboration with other neurohumoral factors that facilitate glucose utilization during ACS. The study population consisted of 1072 consecutive cases with ischemic heart disease who underwent cardiac catheterization (ACS, n = 216; non-ACS, n = 856). Among ACS subjects, biochemical data after acute-phase treatment were available in 91 cases, defined as ACS-remission phase (ACS-rem). Path models based on covariance structure analyses were proposed to clarify the direct contribution of B-type NP (BNP) and noradrenaline to glucose and HOMA-IR levels while eliminating confounding biases. In non-ACS and ACS-rem subjects, although noradrenaline slightly increased glucose and/or HOMA-IR levels (P < 0.03), BNP did not significantly affect them. In contrast, in ACS subjects, high noradrenaline was a significant cause of increases in glucose and HOMA-IR levels (P < 0.001), whereas high BNP was a significant cause of decreases in both parameters (P < 0.005). These findings indicate that BNP and noradrenaline coordinately activate glucose metabolism during ACS, with noradrenaline increasing glucose levels, as an energy substrate, while BNP improves IR and promotes glucose utilization.

## Introduction

Although fatty acid oxidation is the predominant metabolic pathway in the normal adult heart, glycolysis and subsequent glucose metabolism become critical for ATP generation and cardiomyocyte survival during ischemic attack of acute coronary syndrome (ACS)^1–4^. Insulin plays a pivotal role in accelerating the uptake and utilization of glucose in the skeletal muscle as well as in the myocardium. However, we and others have recently reported that insulin resistance (IR) increases in the acute phase of ACS attack^5,6^. The activation of various counter-regulatory neurohumoral factors, such as catecholamines, cortisol and cytokines, during ACS attack antagonizes the action of insulin, which leads to high hepatic glucose output and IR and eventually causes the development of stress-induced hyperglycemia^5,7^.

Natriuretic peptides (NPs) are produced in the heart and classically act on the renal and cardiovascular systems^8^. NPs regulate blood pressure and fluid homeostasis through vasodilatory and diuretic actions and improve cardiac remodeling, mainly through the inhibition of renin-angiotensin-aldosterone and the sympathetic nervous systems^9^. In addition to its classical action of hemodynamic regulation, there has been increasing evidence to indicate that NPs also regulate energy balance and glucose homeostasis^10–14^ as well as thermogenesis in adipose tissues, as recently reported by us and others^15,16^. Furthermore, one study investigating the direct effects of NPs on systemic glucose regulations in humans showed that B-type NP (BNP) administration reduces the plasma glucose concentration^17^. In addition, another *in vitro* study showed that NP increases the glucose uptake during hypoxia in cardiomyocytes^18^. Furthermore, Coué *et al*. very recently reported that NPs promote the uptake of glucose and enhance glucose metabolism in human adipocytes^19^.

Given these previous findings, we hypothesized that the compensatory activations of neurohumoral mechanisms, such as NPs and noradrenaline, facilitate glucose utilization, which overwhelms the IR condition during ACS. One of the difficulties faced in researching the correlations between NPs or noradrenaline and glucose is the intractableness of the statistical approach, as these factors are associated with each other (i.e. correlation between NP and noradrenaline) as well as numerous other factors (i.e. correlations with levels of other neurohumoral factors, degree of obesity or hemodynamics), namely confounding variables. Although there are several ways of eliminating these confounding biases, it is still difficult to control for them if multiple potential confounders are present or if the study population is of insufficient size^20^. A covariance structure analysis is therefore useful for eliminating confounding biases and clarifying possible cause-and-effect relationships^5,20–25^.

Using a covariance structure analysis together with conventional single and multiple regression analyses, this study was designed to evaluate the possible regulation of plasma glucose levels as well as the degree of IR by noradrenaline and BNP in patients with ACS compared to those with non-ACS.

## Results

### The characteristics of the study patients

The clinical characteristics of the 216 cases with ACS and the 856 cases with non-ACS are shown in Table 1. The levels of plasma glucose, insulin and homeostasis model assessment of IR (HOMA-IR) were higher and the potassium lower in the ACS subjects than in the non-ACS subjects, consistent with our previous studies^5,26^. The plasma BNP was only slightly higher, while the plasma noradrenaline was markedly higher in the ACS subjects than in the non-ACS subjects. The LVEF was lower, while the blood pressure was higher in the ACS subjects than in the non-ACS subjects. Among ACS subjects, biochemical data after acute-phase treatment were available in 91 cases, defined as the ACS-remission phase (ACS-rem) (see Methods section). The levels of glucose, insulin, HOMA-IR, BNP and noradrenaline were all decreased during the remission phase of ACS compared to during ischemic attack (Supplementary Table S1).

**Table 1:** Clinical characteristics.

	Overall (n = 1072)	ACS (n = 216)	Non-ACS (n = 856)	P-Value
Age, years old	66 ± 11	61 ± 13	67 ± 11	<0.001
Gender; Male (%)	933 (87.0)	192 (88.9)	741 (86.6)	0.364
BMI, kg/m^2^	24.8 ± 3.7	25.0 ± 4.2	24.7 ± 3.5	0.726
Current smoker (%)	241 (22.5)	74 (34.3)	167 (19.5)	<0.001
BP, mmHg				
Systolic	131 ± 23	134 ± 23	131 ± 23	0.030
Diastolic	70 ± 13	77 ± 13	69 ± 12	<0.001
Mean	95 ± 15	101 ± 15	94 ± 14	<0.001
eGFR, mL/min/1.73 m^2^	70.3 ± 18.4	75.1 ± 19.2	69.0 ± 17.9	<0.001
Na, mmol/L	139.6 ± 2.3	139.1 ± 2.6	139.8 ± 2.2	0.002
K, mmol/L	4.1 ± 0.4	3.9 ± 0.4	4.1 ± 0.3	<0.001
UA, mg/dL	5.9 ± 1.3	5.8 ± 1.4	5.9 ± 1.3	0.355
Glucose, mg/dL	119.8 ± 35.6	142.4 ± 53.3	114.1 ± 26.8	<0.001
Insulin, μU/mL	9.2 ± 10.0	14.4 ± 17.2	7.9 ± 6.6	<0.001
HbA1c, %	6.2 ± 0.9	6.2 ± 1.0	6.2 ± 0.8	0.017
HOMA-IR	3.0 ± 4.7	5.9 ± 8.6	2.3 ± 2.7	<0.001
HOMA- β	63.2 ± 66.5	68.0 ± 57.5	62.0 ± 68.5	0.643
TG, mg/dL	122.8 ± 80.2	114.7 ± 111.4	124.8 ± 70.2	<0.001
LDL-C, mg/dL	101.3 ± 30.3	121.1 ± 34.6	96.3 ± 26.9	<0.001
HDL-C, mg/dL	50.7 ± 14.3	50.4 ± 13.2	50.7 ± 14.5	0.788
BNP, pg/mL	95.6 ± 205.6	96.6 ± 196.5	95.4 ± 208.0	0.042
Noradrenaline, pg/mL	313.5 ± 190.2	473.0 ± 265.3	273.3 ± 139.9	<0.001
LVEF, %	58.1 ± 10.5	54.9 ± 9.6	58.9 ± 10.6	<0.001
Myocardial infarction (%)	—	112 (51.9)	—	—
Unstable angina (%)	—	104 (48.1)	—	—
Diabetes mellitus (%)	405 (37.8)	65 (30.1)	340 (39.7)	0.009
Hypertension (%)	781 (72.9)	130 (60.2)	651 (76.1)	<0.001
*Medication*				
ACE inhibitors (%)	240 (22.4)	13 (6.0)	227 (26.5)	<0.001
ARBs (%)	386 (36.0)	52 (24.1)	334 (39.0)	<0.001
Beta blockers (%)	458 (42.7)	43 (19.9)	415 (48.5)	<0.001
Calcium channel blockers (%)	558 (52.1)	71 (32.9)	487 (56.9)	<0.001
Diuretics (%)	176 (16.4)	18 (8.3)	158 (18.5)	<0.001
Oral antidiabetic agents (%)	274 (25.6)	37 (17.1)	237 (27.7)	0.001

ACE: angiotensin converting enzyme, ACS: Acute Coronary Syndrome, ARBs: angiotensin II type I-receptor blockers, BMI: body mass index, BNP: B-type natriuretic peptide, BP: blood pressure, eGFR: estimated glomerular filtration rate, HbA1c: hemoglobin A1c, HDL-C: high-density lipoprotein, HOMA-β: homeostasis model assessment beta cell function, HOMA-IR: homeostasis model assessment of insulin resistance, K: potassium, LDL-C: low-density lipoprotein, LVEF: left ventricular ejection fraction, Na: sodium, TG: triglycerides, UA: uric acid.

### Simple regression analyses to search for relationships between BNP, noradrenaline and plasma glucose

To evaluate the correlations between the levels of BNP, noradrenaline and plasma glucose, we performed simple regression analyses (Supplementary Fig. S1). A positive correlation between noradrenaline and glucose (P < 0.001) was observed only in the ACS subjects, not in the non-ACS subjects, suggesting that sympathetic nervous system activation by ischemic stress during ACS attack increased plasma glucose levels. Although we observed no significant correlations between BNP and glucose in simple regression analyses, plasma glucose levels tended to be lower in the subjects with higher BNP levels than in those with lower levels between some of the groups (bottom panels in Supplementary Fig. S1). The various confounding factors should be eliminated in order to elucidate the precise relationship between plasma BNP and glucose levels in ischemic heart disease (IHD).

### Multiple regression analyses to determine the factors associated with plasma glucose and HOMA-IR levels

To assess the independent determinants of the plasma glucose (Table 2) as well as HOMA-IR (Table 3) levels, multiple regression analyses were performed in the ACS and non-ACS subjects. The HbA1c was positively correlated with glucose and HOMA-IR in both subjects, as expected. In the ACS subjects, noradrenaline was positively correlated with glucose (P < 0.001) and HOMA-IR (P < 0.001), whereas BNP was negatively correlated with both parameters (P = 0.003 and P = 0.002, respectively). In contrast, in the non-ACS subjects, noradrenaline had relatively little impact on glucose (P = 0.025) and HOMA-IR (P = 0.013), and no significant correlations were noted between BNP and glucose or HOMA-IR. These contrasting effects of noradrenaline and BNP on glucose and HOMA-IR, which were observed only in ACS subjects, were consistently observed, even after various potentially influential clinical factors (Supplementary Tables S2 and S3) as well as pharmacological therapy (Supplementary Tables S4 and S5) were included as the independent variables. On the other hand, the negative correlations between BNP and glucose or HOMA-IR in the ACS subjects were no longer observed during the remission phase, although the positive correlation between noradrenaline and glucose was still observed (Supplementary Tables S6 and S7).

**Table 2:** The multiple regression analyses to identify the clinical factors influencing the plasma glucose level.

ACS R^2^ = 0.451	Non-Standard Coefficient		Standard Regression Coefficient	Test statistic	*P-value*	95% CI	VIF
	Regression Coefficient	Standard Error					
HbA1c	28.084	2.677	0.541	10.490	<*0*.*001*	22.807 to 33.362	1.035
BNP	−0.044	0.015	−0.164	−2.998	*0*.*003*	−0.074 to −0.015	1.166
Noradrenaline	0.069	0.011	0.343	6.173	<*0*.*001*	0.047 to 0.091	1.201
**Non-ACS R**^**2**^ = **0**.**465**	**Non-Standard Coefficient**		**Standard Regression Coefficient**	**Test statistic**	***P-value***	**95% CI**	**VIF**
	**Regression Coefficient**	**Standard Error**					
HbA1c	21.867	0.808	0.679	27.068	<*0*.*001*	20.281 to 23.453	1.002
BNP	−0.005	0.003	−0.041	−1.562	*0*.*119*	−0.012 to 0.001	1.071
Noradrenaline	0.011	0.005	0.058	2.248	*0*.*025*	0.001 to 0.021	1.069

R^2^: adjusted coefficient of determination, CI: confidence interval, VIF: variance inflation factor.

ACS: Acute Coronary Syndrome, BNP: B-type natriuretic peptide, HbA1c: hemoglobin A1c.

**Table 3:** The multiple regression analyses to identify the clinical factors influencing HOMA-IR level.

ACS R^2^ = 0.098	Non-Standard Coefficient		Standard Regression Coefficient	Test statistic	*P-value*	95% CI	VIF
	Regression Coefficient	Standard Error					
HbA1c	1.121	0.554	0.135	2.023	*0*.*044*	0.029 to 2.214	1.036
BNP	−0.010	0.003	−0.217	−3.078	*0*.*002*	−0.016 to −0.004	1.165
Noradrenaline	0.009	0.002	0.276	3.858	<*0*.*001*	0.004 to 0.013	1.198
**Non-ACS R**^**2**^ = **0**.**066**	**Non-Standard Coefficient**		**Standard Regression Coefficient**	**Test statistic**	***P-value***	**95% CI**	**VIF**
	**Regression Coefficient**	**Standard Error**					
HbA1c	0.800	0.107	0.248	7.480	<*0*.*001*	0.590 to 1.010	1.002
BNP	−0.001	0.000	−0.047	−1.357	*0*.*175*	−0.001 to 0.000	1.071
Noradrenaline	0.002	0.001	0.086	2.495	*0*.*013*	0.000 to 0.003	1.070

R^2^: adjusted coefficient of determination, CI: confidence interval, VIF: variance inflation factor.

ACS: Acute Coronary Syndrome, BNP: B-type natriuretic peptide, HbA1c: hemoglobin A1c, HOMA-IR: homeostasis model assessment of insulin resistance.

### Concept of the proposed path model

**Figure 1:**
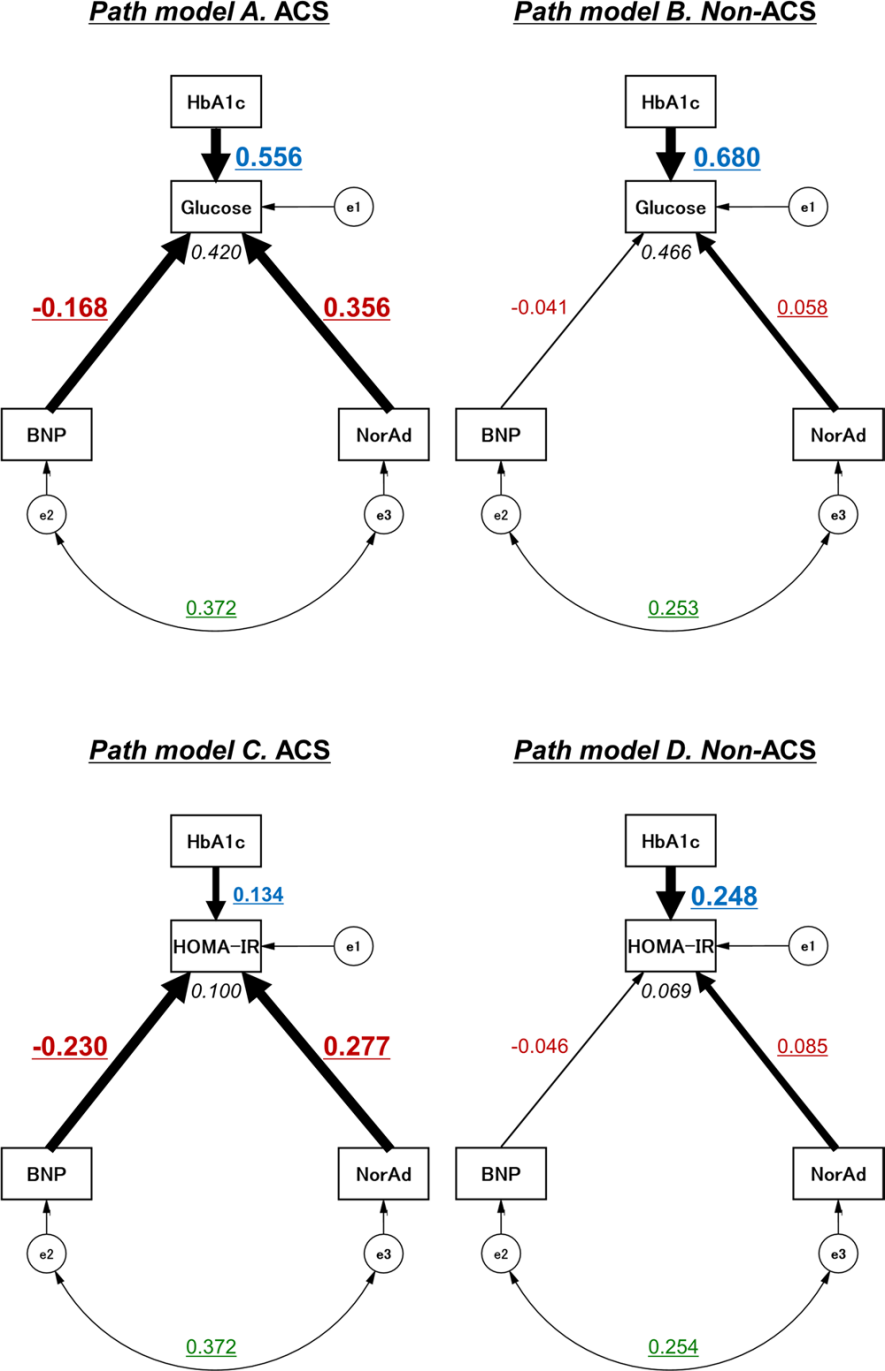
Path diagrams against plasma glucose levels and HOMA-IR levels. Path models theoretically proposed to clarify the contribution of BNP and noradrenaline to glucose as well as to HOMA-IR levels in ACS subjects (n = 216) (*Path model A and C, respectively*) and in non-ACS subjects (n = 856) (*Path model B and D, respectively*). Each path has a coefficient showing the standardized coefficient of a regressing independent variable on a dependent variable of the relevant path. These variables indicate standardized regression coefficients (direct effect) [underlined portions indicate remarkable values], squared multiple correlations [narrow italics] and correlations among exogenous variables [green]. ACS = acute coronary syndrome; BNP = B-type natriuretic peptide; e = extraneous variable; HbA1c = hemoglobin A1c; HOMA-IR, homeostasis model assessment of insulin resistance; NorAd = noradrenaline.

To eliminate the confounding biases and clarify the contribution of noradrenaline and BNP to glucose as well as to HOMA-IR more directly, path models based on a covariance structure analysis were proposed in the ACS, non-ACS (Figs 1 and S2) and ACS-rem groups (Supplementary Fig. S3).

The theoretical path model was created by positioning the levels of BNP and noradrenaline in parallel with consideration of the correlation between these two factors. The association between two factors was linked by two-way arrows. HbA1c and (where indicated) various other clinical parameters were also included as potential factors affecting plasma glucose levels and HOMA-IR. The paths between variables were drawn from independent variables to dependent variables (cause-and-effect relationships) with directional arrows for every regression model.

### Results of the path model

The precise results of the path models are shown in Tables 4 and S8 (ACS and non-ACS subjects), and Supplementary Table S9 (ACS-rem subjects). Consistent with the results from the multiple regression analyses (Tables 2,3 and S6,S7), HbA1c was positively correlated with plasma glucose as well as HOMA-IR levels in all subjects (Figs 1 and S3). Furthermore, BNP and noradrenaline were positively correlated with each other in both ACS and non-ACS subjects (Fig. 1). The exploratory factor analysis with consideration of the positive correlation between BNP and noradrenaline revealed that, in ACS subjects, high noradrenaline levels were a significant cause of increases in glucose levels (Path model A, β = 0.356, P < 0.001) and HOMA-IR (Path model C, β = 0.277, P < 0.001), whereas high BNP levels were a significant cause of decreases in glucose levels (Path model A, β = −0.168, P = 0.003) and HOMA-IR (Path model C, β = −0.230, P = 0.001). Of note, these contrasting influences of noradrenaline and BNP on glucose and HOMA-IR were observed only in ACS subjects; noradrenaline had relatively little impact on glucose (Path model B, β = 0.058, P = 0.024) or HOMA-IR levels (Path model D, β = 0.085, P = 0.013), and BNP no longer affected them (Path models B and D, respectively) in non-ACS subjects. Likewise, these findings of the inverse effects of noradrenaline and BNP on glucose and HOMA-IR, which were observed only in ACS subjects, were consistently observed, even after various potentially influential clinical factors were included in each path model (Path models E-H, Supplementary Fig. S2 and Supplementary Table S8). On the other hand, consistent with the results from the multiple regression analyses (Supplementary Tables S6 and S7), BNP no longer had a significant impact on the glucose or HOMA-IR levels, even during the remission phase, although noradrenaline still increased glucose but not HOMA-IR levels (Supplementary Fig. S3 and Supplementary Table S9).

**Table 4:** The results of path model based on covariance structure analyses.

Clinical Factor			Estimate	Standard error	Test statistic	*P-value*
**Path model (A)**						
Glucose (R^2^ = 0.420)	←	BNP	−0.044	0.015	−3.001	*0*.*003*
	←	Noradrenaline	0.069	0.011	6.339	<*0*.*001*
	←	HbA1c	27.945	2.618	10.675	<*0*.*001*
**Path model (B)**						
Glucose (R^2^ = 0.466)	←	BNP	−0.005	0.003	−1.582	*0*.*114*
	←	Noradrenaline	0.011	0.005	2.253	*0*.*024*
	←	HbA1c	21.875	0.804	27.212	<*0*.*001*
**Path model (C)**						
HOMA-IR (R^2^ = 0.100)	←	BNP	−0.010	0.003	−3.275	*0*.*001*
	←	Noradrenaline	0.009	0.002	3.932	<*0*.*001*
	←	HbA1c	1.113	0.540	2.059	*0*.*039*
**Path model (D)**						
HOMA-IR (R^2^ = 0.069)	←	BNP	−0.001	0.000	−1.345	*0*.*179*
	←	Noradrenaline	0.002	0.001	2.495	*0*.*013*
	←	HbA1c	0.798	0.106	7.501	<*0*.*001*

The results (direct effect) of the path model theoretically proposed analysis to identify the clinical factors influencing the plasma glucose levels or HOMA-IR using ACS subjects (Path models **A** and **C**) and using non-ACS subjects (Path models **B** and **D**) (see Fig. 1).

R^2^: squared multiple correlations.

BNP: B-type natriuretic peptide, HbA1c: hemoglobin A1c, HOMA-IR: homeostasis model assessment of insulin resistance.

**Figure 2:**
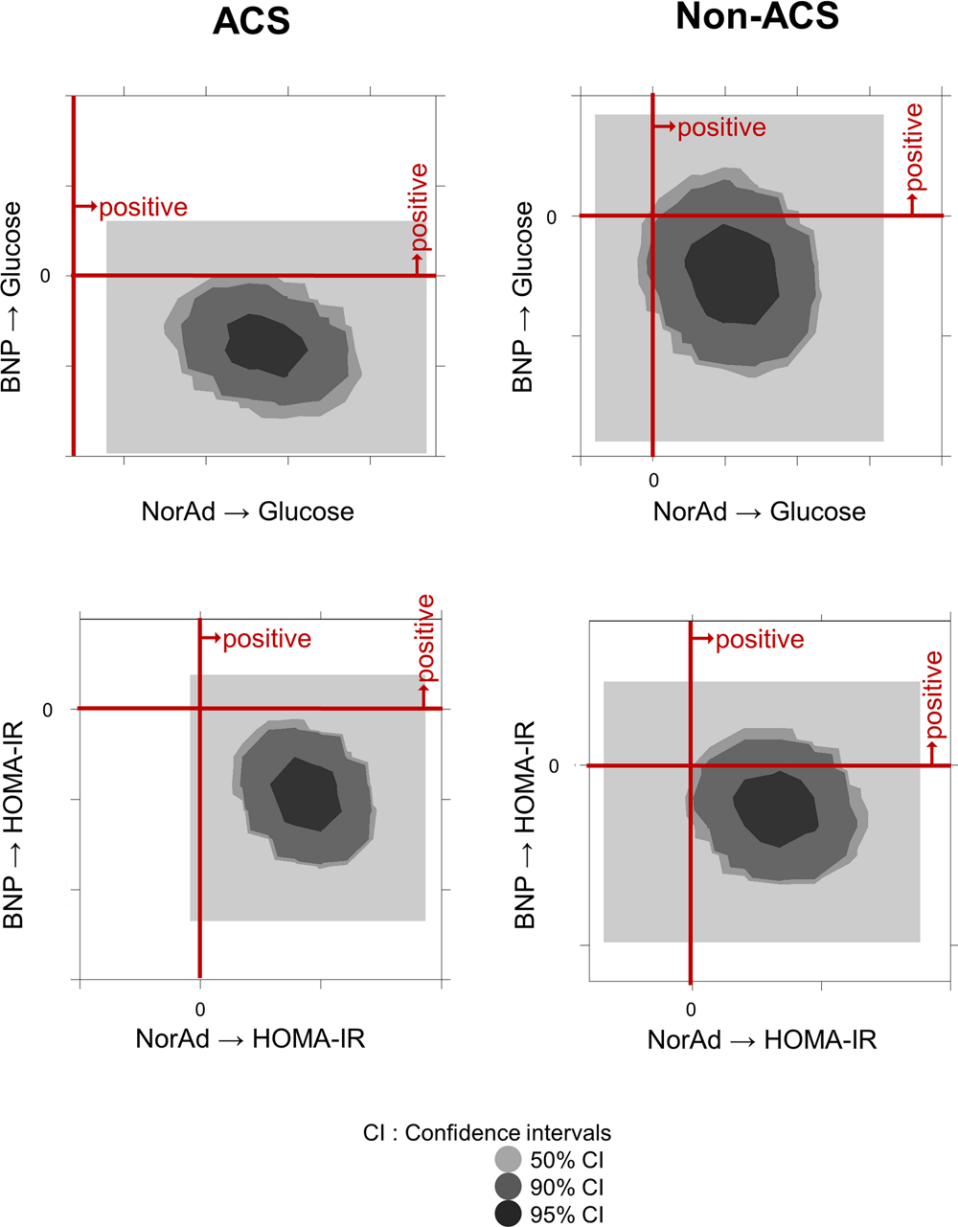
Bayesian estimation. Bivariate marginal posterior distributions are shown in order to help visualize the relationships among pairs of estimands. The credible region (indicated as CI) is conceptually similar to a bivariate confidence region that is familiar to most data analysts acquainted with classical statistical inference methods.

To visualize the relationships among pairs of estimands, bivariate marginal posterior distributions are shown (Fig. 2). The negative regulations of BNP in contrast to the positive regulations of noradrenaline for plasma glucose (upper panels) as well as HOMA-IR (lower panels) levels were more pronounced in ACS subjects (left panels) than in non-ACS (right panels) or ACS-rem subjects (Supplementary Fig. S4).

## Discussion

In addition to the classical actions of NPs on cardiovascular systems, such as hemodynamic regulation, recent studies have indicated that NPs also regulate energy balance and glucose homeostasis^10–12,15,16,19^. Using covariance structure analyses, the present study showed that noradrenaline increases plasma glucose levels (possibly supplying energy substrate), while BNP decreases glucose levels (possibly promoting glucose utilization) and substantially improves IR in ACS subjects but not in non-ACS or ACS-rem subjects. These findings suggest that BNP and noradrenaline act cooperatively to facilitate glucose utilization during an ACS attack, but those collaborative actions become ambiguous during the stable ischemic phase.

Although NPs perform various actions during an ACS attack, the direct effects of NPs on energy metabolism have not yet been clearly proven in humans, because many neurohumoral factors are influenced by one another and contribute to the pathogenesis of cardiovascular diseases. Therefore, the collaborative actions of BNP and noradrenaline on glucose metabolism may be hidden by or become ambiguous due to other confounding factors. The covariance structure analysis used in the present study can be performed based on the cofounding biases and is useful for inferring cause-and-effect relationships^5,20–25^. We successfully proposed a path model based on a covariance structure analysis in order to clarify the causative role of either BNP or noradrenaline in the regulation of plasma glucose and HOMA-IR levels with consideration of the inherent correlation between BNP and noradrenaline themselves. In fact, no significant correlation was observed between BNP and glucose in our simple regression analysis. However, when using a covariance structure analysis, which eliminates potential cofounding factors, the negative correlations between these two factors were clearly delineated only in the ACS subjects (Path models A and C, E and G), with a significant positive correlation preserved between BNP and noradrenaline in both ACS and non-ACS subjects (Figs 1 and S2).

A complex interaction of counter-regulatory hormones, such as catecholamines, cortisol and cytokines can cause the development of stress induced hyperglycemia^5,7^. The derangement of these neurohumoral factors antagonizes the action of insulin, promotes lipolysis, and increases circulating free fatty acid levels, all of which can ultimately lead to high hepatic glucose output and IR^1,5,7^. We recently reported that IR actually increases during ACS attack and suggested that there is an insulin-independent mechanism to promote glucose metabolism in order to cope with the critical ischemic condition^5,26^.

In the meantime, increasing attention is being paid to the possibility that NPs stimulate triglyceride lipolysis, induce adipose tissue browning and activate the thermogenic program^10,13,15,16,27–29^. Furthermore, NPs also promote muscle mitochondrial biogenesis and fat oxidation^12^, all of which lead to protection against glucose intolerance and improve IR. In fact, we found in the present study that BNP substantially improves IR induced by noradrenaline (or other counter-regulatory neurohumoral factors against insulin), particularly during ACS attack (Fig. 1). A recent study by Coué *et al*. proposed biphasic effects of NPs on adipose tissues^19^: NPs first enhance the glucose uptake through the activation of the insulin-independent but guanylyl cyclase-A (GC-A)–cyclic GMP (cGMP)-PKG-Akt-dependent pathway and subsequently promote lipolysis through the same GC-A-cGMP-PKG pathway, as reported previously^13,16,29^. They also reported that the activation of the NP receptor-cGMP pathway in skeletal muscle reduces lipotoxicity and improves the sensitivity of insulin-Akt signaling in IR model mice, although these are relatively chronic effects^11^. The activation of Akt signaling, a major downstream target of insulin, by NPs was also previously reported by others^30,31^. Interestingly, those studies showed that NPs induce Akt signaling activation in cardiomyocytes and exert cardioprotective effects. Previous studies also suggested that NPs improve the glucose metabolism and IR via AMPK signaling pathway in failing heart tissues^29,32^. Meanwhile, we and others reported that NPs reduce oxidative stress and inflammatory activity both *in vitro* and *in vivo*, leading to an improvement of IR^33–35^. Finally, two studies investigated the direct effects of NPs on the regulation of the cardiac glucose metabolism by showing that A-type NP increases myocardial glucose uptake during hypoxia through the upregulation of glucose transporters 1 and 4^18,36^. Thus, in the present study, it is possible that BNP also promoted the uptake of glucose and its metabolism in the myocardium during an ACS attack directly and/or indirectly through the decrease in IR and the improvement of insulin sensitivity. Although the underlying mechanisms by which NPs reduce the glucose level only during the acute phase of an ACS attack remain incompletely understood, the present study at least shows that BNP is able to induce temporary shifts in the whole-body response to glucose and insulin during ACS. Future research is warranted to further explore the mechanistic link between NP signaling and glucose metabolism (such as via a direct effect on skeletal/cardiac muscle, adipose tissue or the liver).

The sample size was somewhat small in the present study. However, a covariance structure analysis as well as a conventional multiple regression analysis consistently showed the contrasting influences of noradrenaline and BNP on glucose and HOMA-IR only in the ACS subjects, with comparable standard regression coefficients. Furthermore, only a limited number of patients actually showed significantly increased BNP levels during ACS in the present study. However, we applied a Bayesian SEM using a program embedded in the IBM SPSS AMOS software program (version 25.0; Amos Development Corporation). Although SEM applies the robust-weighted least-square approach, the Bayesian SEM implements the Gibbs sampler algorithm. Bayesianism permits uncertainty despite little information being available, and Bayesian approaches allow us to incorporate background knowledge into the analyses. We believe that additional testing with Bayesian SEM would be rationalized and helpful for reassessing our data from a completely different angle of statistics. The selected two-dimensional contour line was used in this study because it was easily visualized (Figs 2 and S4).

There is another limitation of this study. Although all patients with ACS in the present study fulfilled the diagnostic criteria, in some cases, the clinical conditions of cardiac catheterization were slightly different between acute myocardial infarction and unstable angina pectoris. Namely, short-term stabilization with medical treatment, such as the use of anti-angina agents, was preferentially performed prior to emergent catheterization in some (but not all) cases, particularly in cases of unstable angina. Thus, the clinical and pathophysiological conditions at the time of emergent catheterization in patients with unstable angina were more diverse than those in patients with acute myocardial infarction, for which coronary angiography tended to be performed in time-sensitive situations. Accordingly, covariance structure analyses using path models A or C revealed that high BNP was a significant cause of the more pronounced decreases that were observed in the glucose and HOMA-IR levels of patients with acute myocardial infarction (path from BNP to glucose; β = −0.224, P = 0.004 and to HOMA-IR; β = −0.266, P = 0.012), whereas the finding that high noradrenaline was a significant cause of the consistent increases in both parameters (path from noradrenaline to glucose; β = 0.306, P < 0.001 and to HOMA-IR; β = 0.217, P = 0.04). These data reinforce the hypothesis that collaborative activities of BNP and noradrenaline are involved in glucose utilization, particularly during the acute phase of an ACS attack.

The chronic persistent action of NPs has been shown to induce adipose tissue browning, lipolysis and thermogenesis as well as muscle mitochondrial biogenesis, which leads to the reduction of IR and the improvement of insulin sensitivity. However, we showed in the present study that the acute action of BNP, in collaboration with noradrenaline, promotes glucose utilization with substantially improved IR in patients with ACS. These findings highlight a previously underappreciated role of NPs in glucose metabolism, which overwhelms the increased IR during ACS attack. The administration of agents that increase circulatory natriuretic peptides levels during the acute phase of ACS may provide therapeutic benefits^37^.

## Methods

### Study patients

Patients with IHD who underwent cardiac catheterization in our institution for the evaluation of coronary organic lesions from May 2015 to March 2018 were included in this study.

IHD was diagnosed based on symptoms, electrocardiography, blood sampling and the coronary artery morphology^20^. Organic lesions producing ≥75% luminal stenosis of the coronary arteries on coronary angiography were defined based on the modified American Heart Association coronary tree segment classification. Patients with coronary spastic angina were also included. ACS was defined as the presence of myocardial infarction (MI) or unstable angina pectoris, as described in detail previously^5,26^. All patients with ACS underwent cardiac catheterization within 24 hours of the onset. Patients were excluded if they were receiving or beginning to receive dialysis, were in cardiogenic shock (Killip grade IV) or had been treated with insulin. Based on these selection criteria, 1072 consecutive cases, including 216 cases with ACS (112 with MI), were enrolled in the present study (Table 1). Among ACS subjects, biochemical data after acute-phase treatment were available in 91 cases (the data were collected mostly at the time of discharge), defined as ACS-rem.

The ethics committee of The Jikei University School of Medicine approved the study protocol (24–355[7121]), and we complied with the routine ethical regulations of our institution. All patients provided their written informed consent before undergoing the procedure, and all clinical investigations were conducted in accordance with the principles expressed in the Declaration of Helsinki. According to our routine ethical regulations, we also posted a notice about the study design and contact information at a public location in our institution.

### Data collection

The clinical characteristics were collected retrospectively from the hospital medical records. We collected blood samples and hemodynamic data during cardiac catheterization. Plasma and serum biochemical analyses, including the levels of glucose, BNP, and noradrenaline, were performed in a central laboratory of our hospital during the study period^5,20^. Some of the patients had comorbid cardiovascular diseases, such as valvular disease, arrhythmia, cardiomyopathy and other conditions. Hypertension, diabetes mellitus (DM) and dyslipidemia were defined as described previously^5,20^. HOMA-IR and the estimated glomerular filtration rate (eGFR) were calculated as described previously^5,20,26^. The left ventricular ejection fraction (LVEF) was measured at the time of left ventriculography^5,20^.

### Statistical analyses

Continuous variables are expressed as the mean ± standard deviation. To compare the clinical characteristics between the ACS and non-ACS subjects, the statistical analyses were performed using the chi-square test for categorial variables and the Mann-Whitney U test for continuous variables. The correlation between the plasma glucose level, BNP and noradrenaline in each group was investigated by a simple regression analysis and expressed as Spearman’s correlation coefficient. Plasma glucose levels were compared among four groups divided by the indicated BNP levels with the Mann-Whitney U test. A multiple regression analysis was performed to compare multiple values. The above-mentioned statistical analyses were performed using the SPSS Statistics software program (version 23.0, SPSS Inc., Chicago, IL, USA). P-values of < 0.05 were considered to indicate statistical significance.

A path analysis based on a covariance structure analysis was used to elucidate the contribution of BNP and noradrenaline to the plasma glucose level and HOMA-IR, respectively, in patients with and without ACS. This analysis compares the power among multiple independent variables that confound each other, and specifically identifies probable causal effects on either the plasma glucose level or HOMA-IR^5,20–25^. The causality model defines some hierarchical regression models between clinical factors and plasma glucose level or HOMA-IR. For every regression, the total variance in the dependent variable is theorized to be caused by either independent variables that are included in the model or by extraneous variables (e)^5,20–25^. The path analysis was performed using the IBM SPSS AMOS software program (version 25; Amos Development Corporation, Meadville, PA, USA). The obtained structural equation models (SEMs) were tested and confirmed at a significance level of P < 0.05.

In addition, we applied a Bayesian SEM using a program embedded in the IBM SPSS AMOS software program (version 25.0; Amos Development Corporation). The frequency polygon was described with the marginal posterior distributions of the estimands. The selected two-dimensional contour line was used in this study because it was easily visualized.

## Supplementary information

Supplementary Information
