# Supplementary material for: Collaborative Activities of Noradrenaline and Natriuretic Peptide for Glucose Utilization in Patients with Acute Coronary Syndrome
Source: Sci Rep. 2019 May 24;9:7822. doi: 10.1038/s41598-019-44216-0 (PMC6534620; doi:10.1038/s41598-019-44216-0)
Supplement: Supplementary file 1 — Supplementary Information [file 41598_2019_44216_MOESM1_ESM.pdf]

Supplementary Information

Collaborative Activities of Noradrenaline and Natriuretic Peptide for Glucose Utilization in Patients with Acute Coronary Syndrome.

Goki Uno[¶], Tomohisa Nagoshi[¶], Akira Yoshii, Yasunori Inoue, Yoshiro Tanaka, Haruka Kimura, Satoshi Ito, Kazuo Ogawa, Toshikazu D. Tanaka, Kosuke Minai, Takayuki Ogawa, Makoto Kawai, Michihiro Yoshimura
[[¶]These authors contributed equally to this work.]

Division of Cardiology, Department of Internal Medicine,
The Jikei University School of Medicine

Supplementary Table S1. Clinical characteristics

	**ACS-rem** (n=91)	**P-Value** vs ACS
Glucose, mg/dL	106.8±20.1	< 0.001
Insulin, μU/mL	9.3±7.6	< 0.001
HOMA-IR	2.5±2.1	< 0.001
BNP, pg/mL	69.1±81.3	0.002
Noradrenaline, pg/mL	282.2±155.8	< 0.001

ACS-rem: remission phase of Acute Coronary Syndrome, BNP: B-type natriuretic peptide, HOMA-IR: homeostasis model assessment of insulin resistance

Supplementary Table S2. The multiple regression analyses to identify the various potential clinical factors influencing the plasma glucose level.

**ACS** R^2=0.446	Non-Standard Coefficient		Standard Regression Coefficient	Test statistic	*P-value*	95% CI	VIF
	Regression Coefficient	Standard Error					
HbA1c	28.971	2.775	0.556	10.439	*<0.001*	23.498 to 34.443	1.080
BNP	-0.043	0.017	-0.150	-2.565	*0.011*	-0.076 to -0.010	1.305
Noradrenaline	0.069	0.012	0.345	5.982	*<0.001*	0.046 to 0.092	1.263
BMI	-0.450	0.700	-0.036	-0.643	*0.521*	-1.831 to 0.931	1.189
TG	-0.023	0.030	-0.043	-0.781	*0.436*	-0.081 to 0.035	1.181
LDL-C	0.012	0.086	0.008	0.137	*0.891*	-0.157 to 0.181	1.179
HDL-C	-0.089	0.230	-0.022	-0.389	*0.698*	-0.542 to 0.364	1.236
Hypertension	-4.661	5.992	-0.043	-0.778	*0.438*	-16.475 to 7.154	1.143

**Non-ACS** R^2=0.468	Non-Standard Coefficient		Standard Regression Coefficient	Test statistic	*P-value*	95% CI	VIF
	Regression Coefficient	Standard Error					
HbA1c	21.595	0.821	0.671	26.308	*<0.001*	19.984 to 23.207	1.038
BNP	-0.004	0.003	-0.030	-1.107	*0.269*	-0.011 to 0.003	1.135
Noradrenaline	0.009	0.005	0.049	1.840	*0.066*	-0.001 to 0.019	1.115
BMI	0.436	0.209	0.057	2.083	*0.038*	0.025 to 0.847	1.214
TG	-0.017	0.010	-0.044	-1.611	*0.107*	-0.037 to 0.004	1.190

LDL-C	0.022	0.026	0.022	0.871	*0.384*	-0.028 to 0.073	1.062
HDL-C	0.028	0.050	0.015	0.554	*0.580*	-0.070 to 0.126	1.170
Hypertension	2.389	1.601	0.038	1.492	*0.136*	-0.753 to 5.530	1.045

R^2: adjusted coefficient of determination, CI: confidence interval, VIF: variance inflation factor.

ACS: Acute Coronary Syndrome, BMI: body mass index, BNP: B-type natriuretic peptide, HbA1c: hemoglobin A1c, HDL-C: high-density lipoprotein, LDL-C: low-density lipoprotein, TG: triglycerides.

Supplementary Table S3. The multiple regression analyses to identify the various potential clinical factors influencing HOMA-IR level.

**ACS** R^2=0.147	Non-Standard Coefficient		Standard Regression Coefficient	Test statistic	*P-value*	95% CI	VIF
	Regression Coefficient	Standard Error					
HbA1c	0.812	0.554	0.097	1.466	*0.144*	-0.280 to 1.905	1.081
BNP	-0.007	0.003	-0.157	-2.161	*0.032*	-0.014 to -0.001	1.305
Noradrenaline	0.008	0.002	0.247	3.448	*0.001*	0.003 to 0.013	1.263
BMI	0.496	0.140	0.247	3.550	*<0.001*	0.220 to 0.771	1.189
TG	0.000	0.006	0.001	0.017	*0.986*	-0.012 to 0.012	1.181
LDL-C	0.012	0.017	0.047	0.681	*0.497*	-0.022 to 0.045	1.178
HDL-C	-0.029	0.046	-0.046	-0.644	*0.520*	-0.120 to 0.061	1.236
Hypertension	-0.293	1.196	-0.017	-0.245	*0.806*	-2.653 to 2.066	1.144

**Non-ACS** R^2=0.100	Non-Standard Coefficient		Standard Regression Coefficient	Test statistic	*P-value*	95% CI	VIF
	Regression Coefficient	Standard Error					
HbA1c	0.686	0.107	0.213	6.412	*<0.001*	0.476 to 0.896	1.038
BNP	0.000	0.000	-0.019	-0.540	*0.590*	-0.001 to 0.001	1.137
Noradrenaline	0.002	0.001	0.095	2.765	*0.006*	0.001 to 0.003	1.116
BMI	0.112	0.027	0.148	4.120	*<0.001*	0.059 to 0.166	1.214
TG	0.003	0.001	0.067	1.879	*0.061*	0.000 to 0.005	1.190

LDL-C	0.002	0.003	0.019	0.563	*0.574*	-0.005 to 0.008	1.061
HDL-C	-0.008	0.007	-0.045	-1.273	*0.203*	-0.021 to 0.004	1.170
Hypertension	0.138	0.209	0.022	0.660	*0.509*	-0.272 to 0.547	1.044

R^2: adjusted coefficient of determination, CI: confidence interval, VIF: variance inflation factor.

ACS: Acute Coronary Syndrome, BMI: body mass index, BNP: B-type natriuretic peptide, HbA1c: hemoglobin A1c, HDL-C: high-density lipoprotein, HOMA-IR: homeostasis model assessment of insulin resistance, LDL-C: low-density lipoprotein, TG: triglycerides.

Supplementary Table S4. The multiple regression analyses to identify the clinical factors including pharmacological therapy influencing the plasma glucose level.

**ACS** R^2=0.465	Non-Standard Coefficient		Standard Regression Coefficient	Test statistic	*P-value*	95% CI	VIF
	Regression Coefficient	Standard Error					
HbA1c	26.925	3.207	0.519	8.395	*<0.001*	20.601 to 33.249	1.523
BNP	-0.040	0.015	-0.147	-2.617	*0.010*	-0.069 to -0.010	1.247
Noradrenaline	0.069	0.011	0.343	6.151	*<0.001*	0.047 to 0.091	1.241
ACE inhibitors	27.812	12.490	0.125	2.227	*0.027*	3.186 to 52.438	1.253
ARBs	-6.748	7.407	-0.054	-0.911	*0.363*	-21.352 to 7.855	1.401
Beta blockers	-19.088	7.725	-0.143	-2.471	*0.014*	-34.319 to -3.857	1.325
CCBs	-1.400	6.877	-0.012	-0.204	*0.839*	-14.960 to 12.160	1.465
Diuretics	-5.574	10.237	-0.029	-0.544	*0.587*	-25.759 to 14.610	1.136
OHAs	8.237	8.659	0.059	0.951	*0.343*	-8.836 to 25.310	1.509

**Non-ACS** R^2=0.483	Non-Standard Coefficient		Standard Regression Coefficient	Test statistic	*P-value*	95% CI	VIF
	Regression Coefficient	Standard Error					
HbA1c	18.459	1.002	0.574	18.430	*<0.001*	16.493 to 20.425	1.594
BNP	-0.006	0.003	-0.049	-1.842	*0.066*	-0.013 to 0.000	1.142
Noradrenaline	0.010	0.005	0.053	1.997	*0.046*	0.000 to 0.020	1.140
ACE inhibitors	-1.348	1.729	-0.022	-0.779	*0.436*	-4.741 to 2.046	1.344

ARBs	0.420	1.578	0.008	0.266	*0.790*	-2.677 to 3.516	1.362
Beta blockers	0.913	1.371	0.017	0.666	*0.505*	-1.777 to 3.604	1.080
CCBs	0.100	1.384	0.002	0.072	*0.943*	-2.617 to 2.817	1.083
Diuretics	1.483	1.852	0.021	0.801	*0.423*	-2.152 to 5.117	1.186
OHAs	10.643	1.855	0.178	5.737	*<0.001*	7.002 to 14.284	1.577

R^2: adjusted coefficient of determination, CI: confidence interval, VIF: variance inflation factor.

ACE: angiotensin converting enzyme, ACS: Acute Coronary Syndrome, ARBs: angiotensin II type I-receptor blockers, BNP: B-type natriuretic peptide, CCBs: calcium channel blockers, HbA1c: hemoglobin A1c, OHAs: oral hypoglycemic agents.

Supplementary Table S5. The multiple regression analyses to identify the clinical factors including pharmacological therapy influencing HOMA-IR level.

**ACS** R^2=0.094	Non-Standard Coefficient		Standard Regression Coefficient	Test statistic	*P-value*	95% CI	VIF
	Regression Coefficient	Standard Error					
HbA1c	1.136	0.675	0.136	1.682	*0.094*	-0.196 to 2.467	1.530
BNP	-0.010	0.003	-0.219	-2.961	*0.003*	-0.017 to -0.003	1.275
Noradrenaline	0.009	0.002	0.268	3.679	*<0.001*	0.004 to 0.013	1.237
ACE inhibitors	4.544	2.619	0.127	1.735	*0.084*	-0.619 to 9.708	1.255
ARBs	0.369	1.557	0.018	0.237	*0.813*	-2.701 to 3.440	1.390
Beta blockers	-1.592	1.622	-0.074	-0.982	*0.327*	-4.790 to 1.606	1.329
CCBs	1.485	1.444	0.081	1.028	*0.305*	-1.362 to 4.331	1.455
Diuretics	-1.945	2.152	-0.063	-0.904	*0.367*	-6.188 to 2.298	1.144
OHAs	-0.046	1.817	-0.002	-0.026	*0.980*	-3.629 to 3.536	1.512

**Non-ACS** R^2=0.069	Non-Standard Coefficient		Standard Regression Coefficient	Test statistic	*P-value*	95% CI	VIF
	Regression Coefficient	Standard Error					
HbA1c	0.928	0.135	0.288	6.889	*<0.001*	0.664 to 1.193	1.593
BNP	-0.001	0.000	-0.059	-1.664	*0.097*	-0.002 to 0.000	1.142
Noradrenaline	0.001	0.001	0.077	2.160	*0.031*	0.000 to 0.003	1.142
ACE inhibitors	0.215	0.233	0.035	0.922	*0.357*	-0.243 to 0.672	1.344

ARBs	-0.004	0.213	-0.001	-0.019	*0.985*	-0.421 to 0.413	1.362
Beta blockers	0.177	0.185	0.033	0.957	*0.339*	-0.186 to 0.539	1.080
CCBs	-0.163	0.187	-0.030	-0.873	*0.383*	-0.529 to 0.203	1.081
Diuretics	0.248	0.249	0.036	0.994	*0.321*	-0.242 to 0.737	1.188
OHAs	-0.415	0.250	-0.069	-1.662	*0.097*	-0.905 to 0.075	1.576

R^2: adjusted coefficient of determination, CI: confidence interval, VIF: variance inflation factor.
ACE: angiotensin converting enzyme, ACS: Acute Coronary Syndrome, ARBs: angiotensin II type I-receptor blockers, BNP: B-type natriuretic peptide, CCBs: calcium channel blockers, HbA1c: hemoglobin A1c, HOMA-IR: homeostasis model assessment of insulin resistance, OHAs: oral hypoglycemic agents.

Supplementary Table S6. The multiple regression analyses to identify the clinical factors influencing the plasma glucose level.

**ACS-rem** R^2=0.324	Non-Standard Coefficient		Standard Regression Coefficient	Test statistic	*P-value*	95% CI	VIF
	Regression Coefficient	Standard Error					
HbA1c	8.820	1.679	0.462	5.253	*<0.001*	5.483 to 12.157	1.028
BNP	-0.028	0.021	-0.113	-1.303	*0.196*	-0.071 to 0.015	1.001
Noradrenaline	0.036	0.011	0.283	3.219	*0.002*	0.014 to 0.059	1.027

R^2: adjusted coefficient of determination, CI: confidence interval, VIF: variance inflation factor.
ACS-rem: remission phase of Acute Coronary Syndrome, BNP: B-type natriuretic peptide, HbA1c: hemoglobin A1c.

Supplementary Table S7. The multiple regression analyses to identify the clinical factors influencing HOMA-IR level.

**ACS-rem** R^2=0.074	Non-Standard Coefficient		Standard Regression Coefficient	Test statistic	*P-value*	95% CI	VIF
	Regression Coefficient	Standard Error					
HbA1c	0.453	0.206	0.226	2.197	*0.031*	0.043 to 0.863	1.028
BNP	-0.004	0.003	-0.145	-1.431	*0.156*	-0.009 to 0.001	1.001
Noradrenaline	0.002	0.001	0.150	1.463	*0.147*	-0.001 to 0.005	1.027

R^2: adjusted coefficient of determination, CI: confidence interval, VIF: variance inflation factor.

ACS-rem: remission phase of Acute Coronary Syndrome, BNP: B-type natriuretic peptide, HbA1c: hemoglobin A1c, HOMA-IR: homeostasis model assessment of insulin resistance

Supplementary Table S8. The results of path model based on covariance structure analyses.

Clinical Factor			Estimate	Standard error	Test statistic	*P-value*
Path model (E)						
Glucose (R^2 =0.429)	←	BNP	-0.049	0.015	-3.361	*<0.001*
	←	Noradrenaline	0.071	0.011	6.514	*< 0.001*
	←	HbA1c	28.602	2.672	10.705	*< 0.001*
	←	BMI	-0.532	0.676	-0.787	*0.431*
	←	TG	-0.023	0.025	-0.922	*0.356*
	←	LDL-C	0.008	0.082	0.093	*0.926*
	←	HDL-C	-0.101	0.222	-0.455	*0.649*
	←	Hypertension	-4.717	5.779	-0.816	*0.414*
Path model (F)						
Glucose (R^2 =0.472)	←	BNP	-0.004	0.003	-1.173	*0.241*
	←	Noradrenaline	0.009	0.005	1.887	*0.059*
	←	HbA1c	21.604	0.815	26.516	*< 0.001*
	←	BMI	0.421	0.202	2.078	*0.038*
	←	TG	-0.016	0.010	-1.584	*0.113*
	←	LDL-C	0.021	0.025	0.818	*0.413*
	←	HDL-C	0.029	0.049	0.600	*0.548*
	←	Hypertension	2.439	1.567	1.556	*0.120*
Path model (G)						
HOMA-IR (R^2 =0.153)	←	BNP	-0.007	0.003	-2.450	*0.014*
	←	Noradrenaline	0.008	0.002	3.651	*< 0.001*
	←	HbA1c	0.813	0.533	1.526	*0.127*
	←	BMI	0.496	0.135	3.676	*<0.001*
	←	TG	0.000	0.005	0.058	*0.954*
	←	LDL-C	0.012	0.016	0.727	*0.467*
	←	HDL-C	-0.029	0.044	-0.653	*0.514*
	←	Hypertension	-0.312	1.152	-0.271	*0.786*
Path model (H)						
HOMA-IR (R^2 =0.110)	←	BNP	0.000	0.000	-0.546	*0.585*
	←	Noradrenaline	0.002	0.001	2.835	*0.005*
	←	HbA1c	0.683	0.106	6.438	*< 0.001*
	←	BMI	0.112	0.026	4.252	*< 0.001*
	←	TG	0.003	0.001	1.899	*0.058*
	←	LDL-C	0.002	0.003	0.589	*0.556*
	←	HDL-C	-0.008	0.006	-1.314	*0.189*
	←	Hypertension	0.134	0.204	0.657	*0.511*

The results (direct effect) of the path model theoretically proposed analysis to identify the clinical factors influencing the plasma glucose levels or HOMA-IR using ACS subjects (**Path models E** and **G**) and using non-ACS subjects (**Path models F** and **H**) (see Supplementary Fig. S2).
R^2: squared multiple correlations.

BMI: body mass index, BNP: B-type natriuretic peptide, HbA1c: hemoglobin A1c, HDL-C: high-density lipoprotein, HOMA-IR: homeostasis model assessment of insulin resistance, LDL-C: low-density lipoprotein, TG: triglycerides.

Supplementary Table S9. The results of path model based on covariance structure analyses.

Clinical Factor			Estimate	Standard error	Test statistic	*P-value*
Path model (I)						
Glucose (R^2 =0.319)	←	BNP	-0.028	0.021	-1.326	*0.185*
	←	Noradrenaline	0.036	0.011	3.318	< *0.001*
	←	HbA1c	8.820	1.628	5.416	< *0.001*
Path model (J)						
HOMA-IR (R^2 =0.096)	←	BNP	-0.004	0.003	-1.456	*0.145*
	←	Noradrenaline	0.002	0.001	1.508	*0.132*
	←	HbA1c	0.453	0.200	2.265	*0.024*

The results (direct effect) of the path model theoretically proposed analysis to identify the clinical factors influencing the plasma glucose level or HOMA-IR using ACS-rem subjects (**Path models I** and **J**) (see Supplementary Fig. S3).
R^2: squared multiple correlations.
BNP: B-type natriuretic peptide, HbA1c: hemoglobin A1c, HOMA-IR: homeostasis model assessment of insulin resistance.

Supplementary Figure Legends

Supplementary Figure S1. Associations between BNP, noradrenaline and plasma glucose levels.

The simple regression analyses between BNP and plasma glucose (top panel), between noradrenaline and plasma glucose (middle panel) in ACS subjects (left side) and in non-ACS subjects (right side) are shown. Comparison of plasma glucose levels among four groups divided by indicated BNP levels in ACS (left side) and in non-ACS (right side) are shown in the bottom panels.

ACS = Acute Coronary Syndrome; BNP = B-type natriuretic peptide

Supplementary Figure S2. Path diagrams against plasma glucose and HOMA-IR levels (including various potential clinical factors).

Path models theoretically proposed to clarify the contribution of various potential clinical factors to glucose as well as to HOMA-IR levels in ACS subjects (n=216) (***Path model E** and **G**, respectively*) and in non-ACS subjects (n=856) (***Path model F** and **H**, respectively*). Each path has a coefficient showing the standardized coefficient of a regressing independent variable on a dependent variable of the relevant path. These variables indicate standardized regression coefficients (direct effect) [underlined portions indicate remarkable values], squared multiple correlations [narrow italics] and correlations among exogenous variables [green]. ACS = acute coronary syndrome; BMI = body mass index; BNP = B-type natriuretic peptide; e = extraneous variable; HbA1c = hemoglobin A1c; HDL-C = high-density lipoprotein; HOMA-IR, homeostasis model assessment of insulin resistance; HTN = hypertension; LDL-C = low-density lipoprotein; NorAd = noradrenaline; TG = triglycerides.

Supplementary Figure S3. Path diagrams against plasma glucose and HOMA-IR levels in ACS-rem subjects.

Path models theoretically proposed to clarify a contribution of BNP and noradrenaline to either plasma glucose levels (***Path model I***) or HOMA-IR levels (***Path model J***) in ACS-remission phase subjects (n=91). Each path has a coefficient showing the standardized coefficient of a regressing independent variable on a dependent variable of the relevant path. These variables indicate standardized regression coefficients (direct effect) [underlined portions indicate remarkable values], squared multiple correlations [narrow italics] and correlations among exogenous variables [green].

ACS-rem = remission phase of Acute Coronary Syndrome; BNP = B-type natriuretic peptide; e = extraneous variable; HbA1c = hemoglobin A1c; HOMA-IR, homeostasis model assessment of insulin resistance; NorAd = noradrenaline

Supplementary Figure S4. Bayesian estimation.

Bivariate marginal posterior distributions are shown to visualize the relationships among pairs of estimands. A credible region (indicated as CI) is conceptually similar to a bivariate confidence region that is familiar to most data analysts acquainted with classical statistical inference methods.

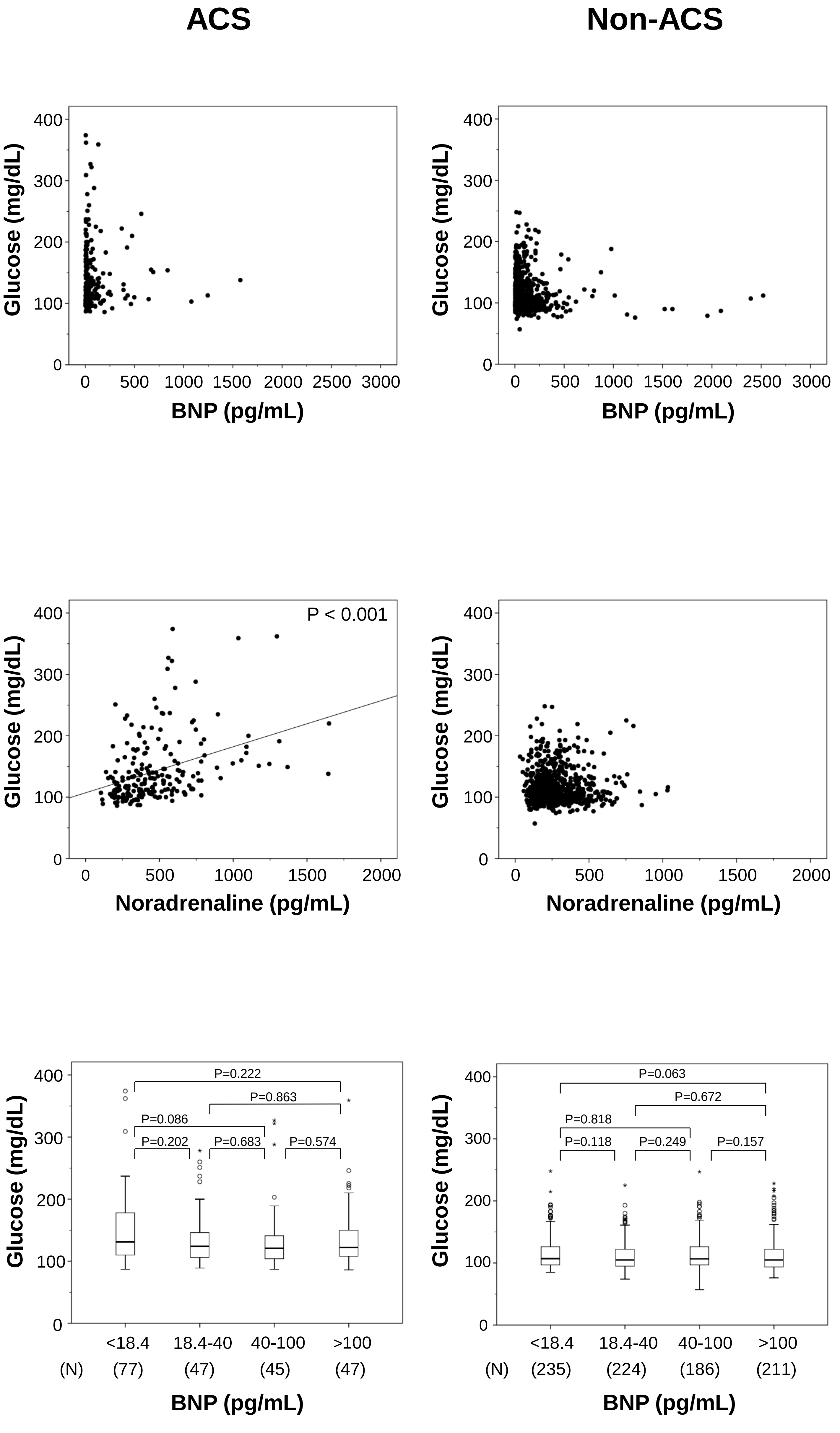

<u>Supplementary Figure S1</u>

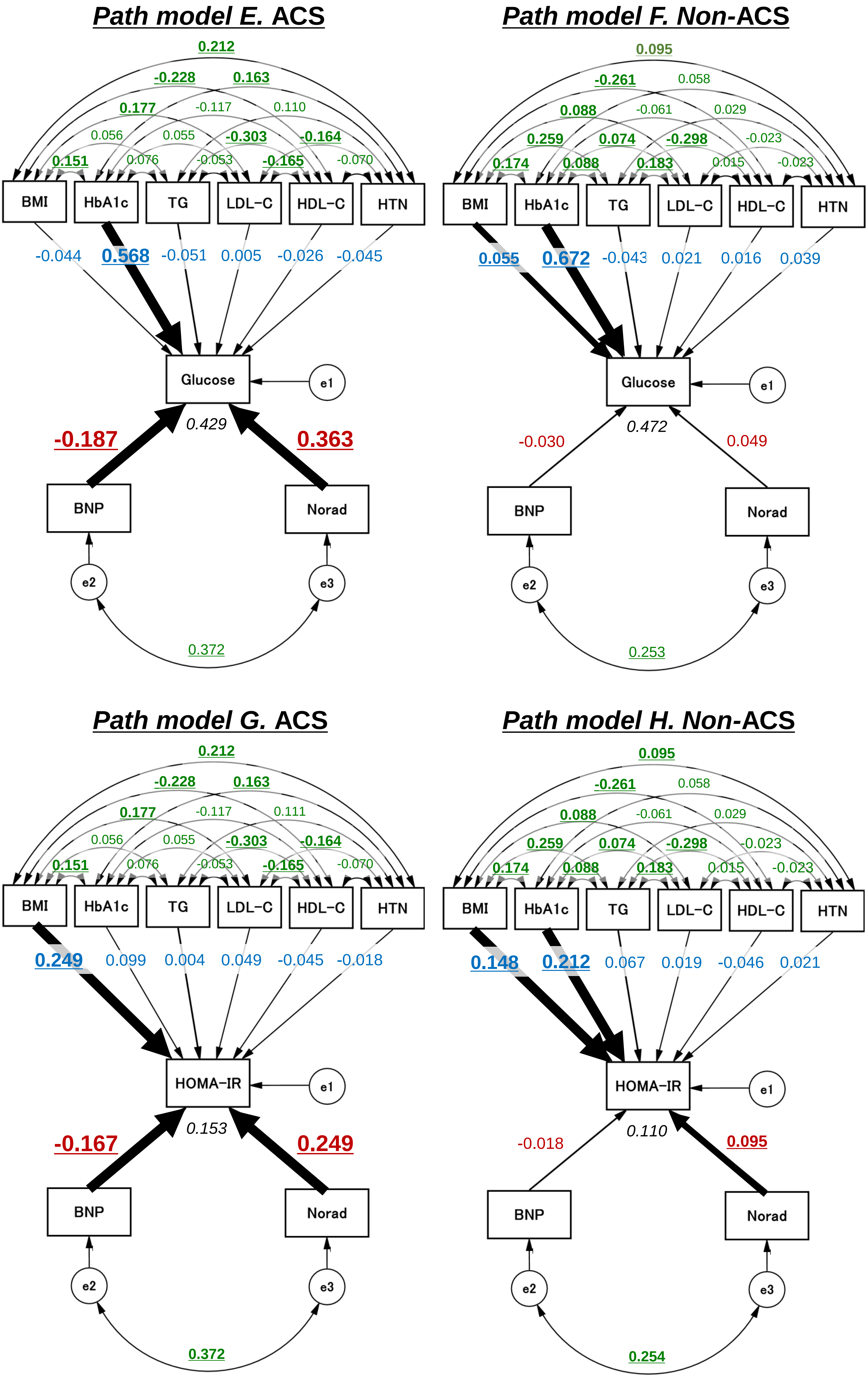

Supplemental Figure S2

Path model I. ACS-rem

Path model J. ACS-rem

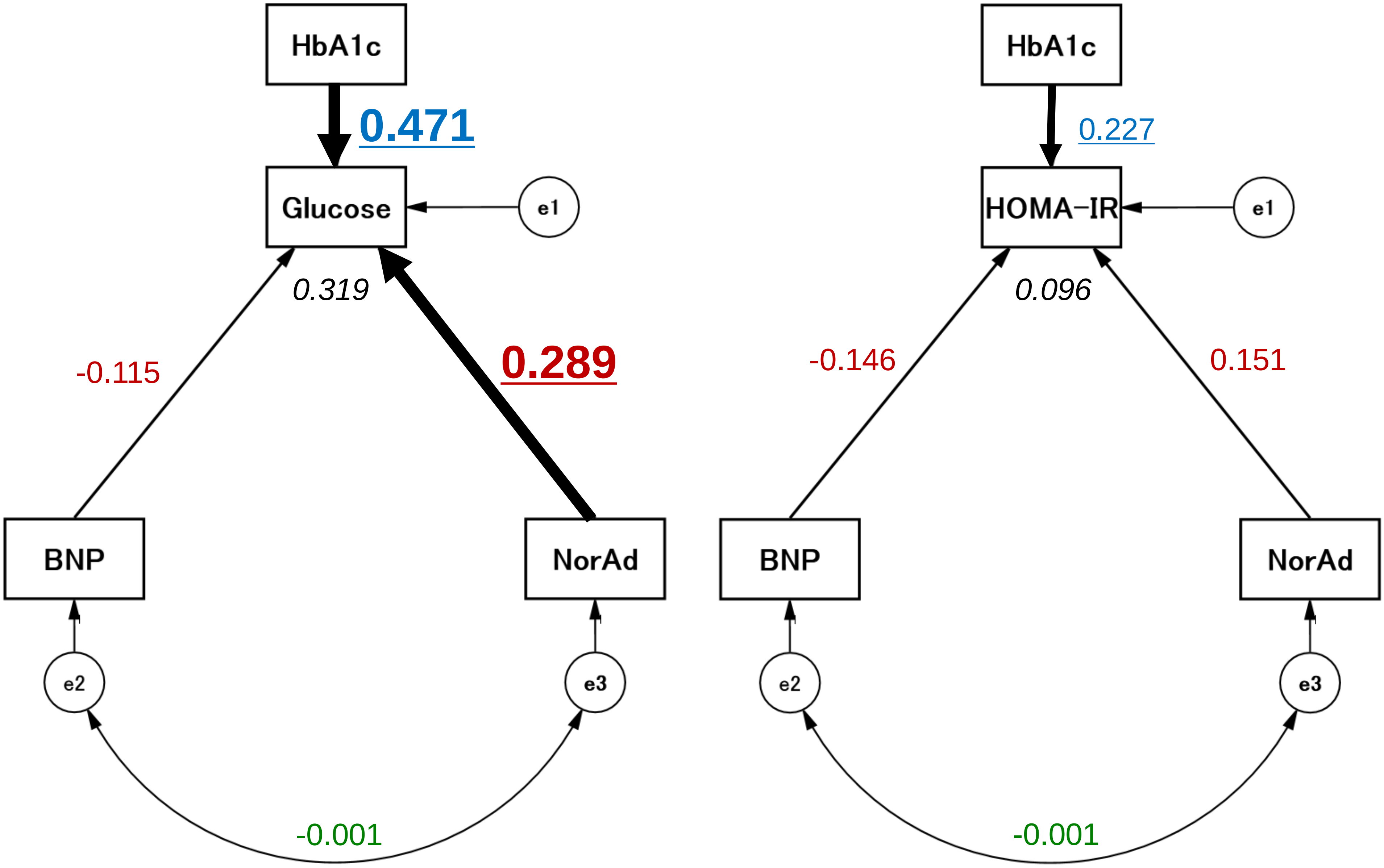

Supplementary Figure S3

ACS-rem

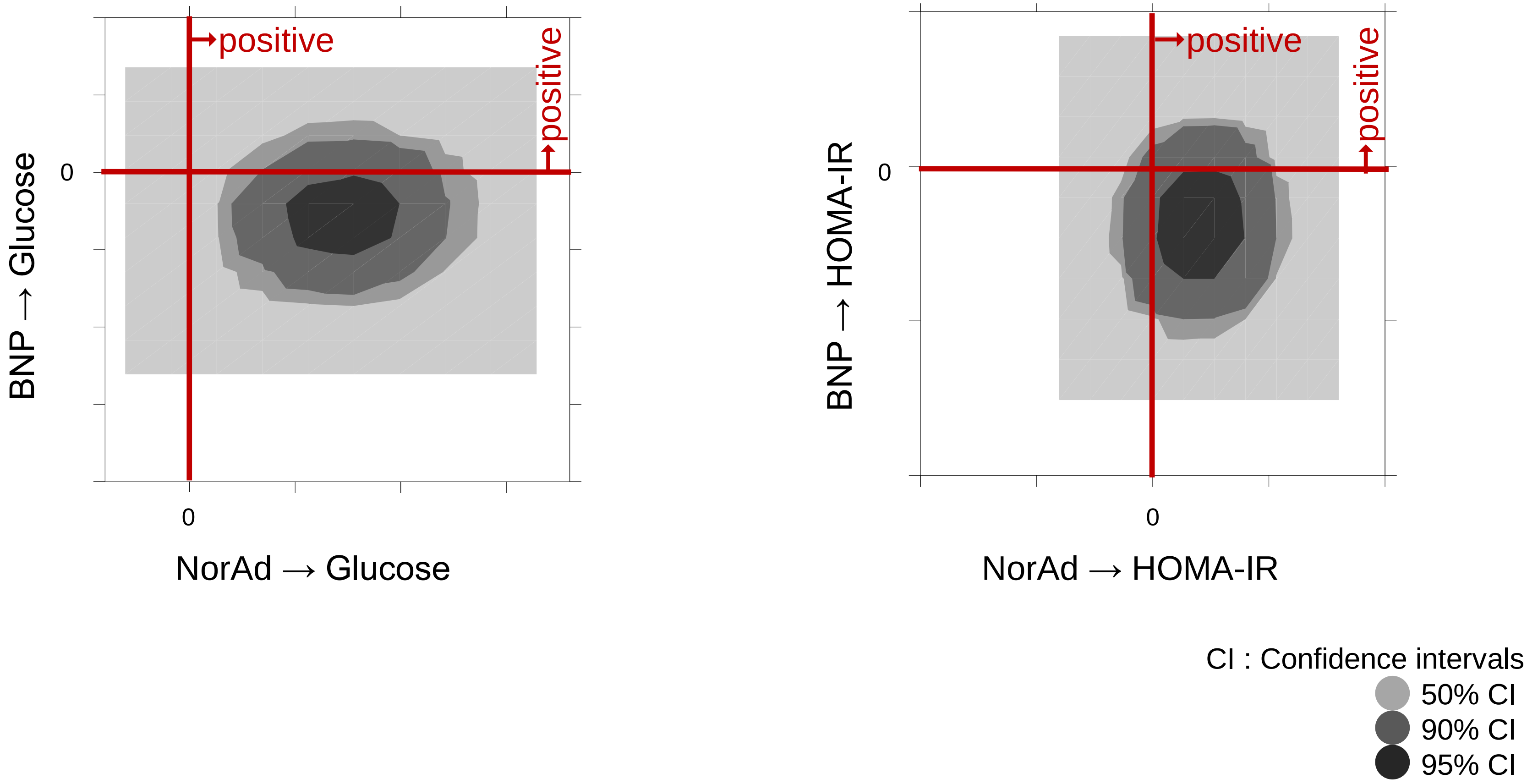

Supplementary Figure S4
